# (1*S*,3*R*,8*R*)-2,2-Dibromo-10-bromo­methyl-3,7,7-trimethyl­tricyclo­[6.4.0.0^1,3^]dodec-9-ene

**DOI:** 10.1107/S1600536812046430

**Published:** 2012-11-24

**Authors:** Abdelouahd Oukhrib, Ahmed Benharref, Mohamed Saadi, Jean-Claude Daran, Moha Berraho

**Affiliations:** a Laboratoire de Chimie des Substances Naturelles, "Unité Associé au CNRST (URAC16)", Faculté des Sciences Semlalia, BP 2390 Bd My Abdellah, 40000 Marrakech, Morocco; b Laboratoire de Chimie du Solide Appliquée, Faculté des Sciences, Avenue Ibn Battouta, BP 1014 Rabat, Morocco; c Laboratoire de Chimie de Coordination, 205 route de Narbonne, 31077 Toulouse Cedex 04, France

## Abstract

The title compound, C_16_H_23_Br_3_, was synthesized from β-himachalene (3,5,5,9-tetra­methyl-2,4a,5,6,7,8-hexa­hydro-1*H*-benzocyclo­heptene), which was isolated from the essential oil of the Atlas cedar (*Cedrus Atlantica*). The mol­ecule is built up from fused six- and seven-membered rings and an additional three-membered ring from the reaction of himachalene with dibromo­carbene. The six-membered ring has an envelope conformation (the flap atom being the C atom shared with the three-membered ring, whereas the seven-membered ring displays a screw boat conformation; the dihedral angle between the rings (defined by the near coplanar atoms) is 56.5 (2)°.

## Related literature

For the isolation of β-himachalene, see: Joseph & Dev (1968 ▶); Plattier & Teiseire (1974 ▶). For the reactivity of this sesquiterpene, see: Lassaba *et al.* (1997 ▶); Chekroun *et al.* (2000 ▶); El Jamili *et al.* (2002 ▶); Sbai *et al.* (2002 ▶); Dakir *et al.* (2004 ▶); Benharref *et al.* (2010 ▶). For its biological activity, see: Daoubi *et al.* (2004 ▶). For conformational analysis, see: Cremer & Pople (1975 ▶).
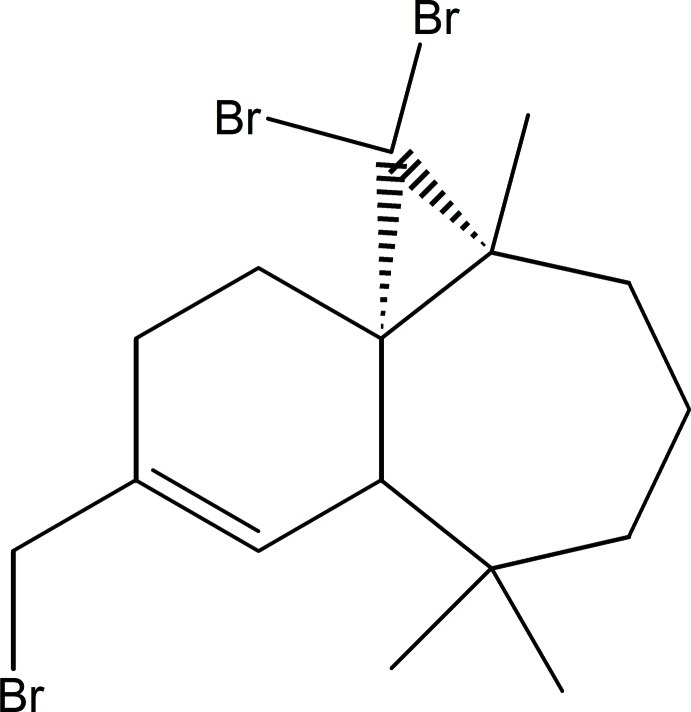


## Experimental

### 

#### Crystal data

C_16_H_23_Br_3_
*M* *_r_* = 455.07
Orthorhombic, 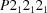

*a* = 9.2614 (5) Å
*b* = 12.8215 (8) Å
*c* = 14.3966 (11) Å
*V* = 1709.52 (19) Å^3^
*Z* = 4
Mo *K*α radiation
μ = 7.07 mm^−1^
*T* = 180 K
0.49 × 0.31 × 0.08 mm

#### Data collection

Agilent Xcalibur (Sapphire1, long nozzle) diffractometer
Absorption correction: multi-scan (*CrysAlis PRO*; Agilent, 2010 ▶) *T*_min_ = 0.135, *T*_max_ = 1.000
9721 measured reflections
3461 independent reflections
3121 reflections with *I* > 2σ(*I*)
*R*_int_ = 0.049

#### Refinement

*R*[*F* ^2^ > 2σ(*F* ^2^)] = 0.034
*wR*(*F* ^2^) = 0.075
*S* = 1.04
3461 reflections
176 parameters
H-atom parameters constrained
Δρ_max_ = 0.66 e Å^−3^
Δρ_min_ = −0.55 e Å^−3^
Absolute structure: Flack (1983 ▶), 1460 Friedel pairs
Flack parameter: 0.012 (16)

### 

Data collection: *CrysAlis PRO* (Agilent, 2010 ▶); cell refinement: *CrysAlis PRO*; data reduction: *CrysAlis PRO*; program(s) used to solve structure: *SHELXS97* (Sheldrick, 2008 ▶); program(s) used to refine structure: *SHELXL97* (Sheldrick, 2008 ▶); molecular graphics: *ORTEP-3 for Windows* (Farrugia, 2012 ▶); software used to prepare material for publication: *WinGX* (Farrugia, 2012 ▶).

## Supplementary Material

Crystal structure: contains datablock(s) I, global. DOI: 10.1107/S1600536812046430/im2411sup1.cif

Structure factors: contains datablock(s) I. DOI: 10.1107/S1600536812046430/im2411Isup2.hkl

Supplementary material file. DOI: 10.1107/S1600536812046430/im2411Isup3.cml

Additional supplementary materials:  crystallographic information; 3D view; checkCIF report

## Figures and Tables

**Fig. 1.**
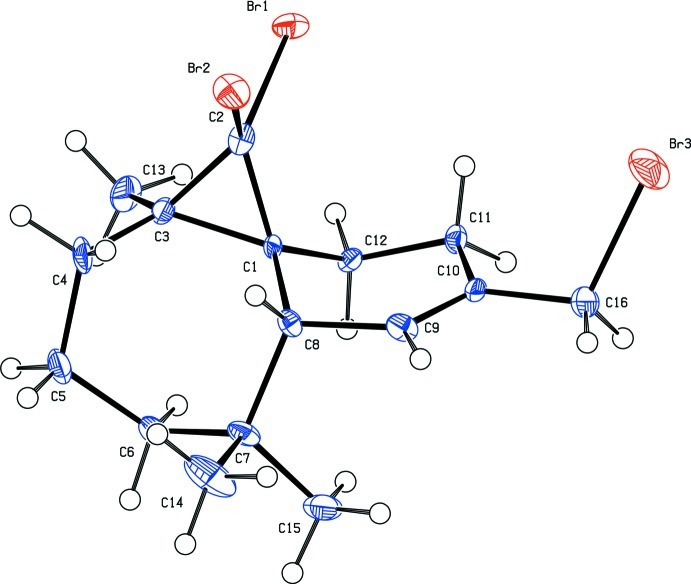
Molecular structure of the title compound with displacement ellipsoids drawn at the 30% probability. level. H atoms are represented as small spheres of arbitrary radii.

## supplementary crystallographic information

### Comment

Our work lies within the framework of the valorization of the most abundant essential oils in Morocco, such as the one from *Cedrus atlantica*. This oil is made up mainly (75%) of bicyclic sesquiterpenes hydrocarbons, among which is found β-himachalene (Joseph & Dev, 1968; Plattier & Teiseire, 1974). The reactivity of this sesquiterpene and its derivatives has been studied extensively by our team in order to prepare new products having biological proprieties (Lassaba *et al.*, 1997; Chekroun *et al.*, 2000; El Jamili *et al.*, 2002; Sbai *et al.*, 2002; Dakir *et al.*, 2004; Benharref *et al.*,2010). Indeed, these compounds were tested, using the food poisoning technique, for their potential antifungal activity against the phytopathogen *Botrytis cinerea* (Daoubi *et al.*, 2004). In a previous work (El Jamili *et al.*, 2002) we have prepared (1*S*,3*R*,8*R*)-2,2-dibromo- 3,7,7,10- tetramethyltricyclo [6.4.0.01,3] dodec-9-ene from β-himachalene, which by treatment with *N*-bromosuccinimide gave the title compound. The structure of this new product was determined by single-crystal X-ray structure analysis. The molecule is built up from two fused six-and seven- membered rings and an additional three-membered ring from the reaction with the carbene (Fig.1). The six-membered ring has an envelope conformation, as indicated by the total puckering amplitude QT = 0.497 (4) Å and spherical polar angle θ= 129.5 (5)° with φ = 149.2 (7)°, whereas the seven-membered ring displays a screw boat conformation with QT = 1.1556 (4) Å, θ = 88.2 (20)°, φ2 = -48.26 (20)° and φ3 = -117.47 (7)° (Cremer & Pople, 1975). Owing to the presence of Br atoms, the absolute configuration could be fully confirmed as C1(S), C3(*R*)and C8(*R*) by refining the Flack (1983) parameter as C1(S), C3(*R*)and C8(*R*).

### Experimental

In a reactor containing a solution of (1*S*,3*R*,8*R*)-2,2-dibromo-3,7,7,10 tetramethyltricyclo[6.4.0.0_1_,_3_] dodec-9-ene (1 g, 2.6 mmol) in 50 ml of tetrahydrofuran and water (THF/H_2_O) (4:1) cooled to 273 K and kept in the dark, was added in small portions 1 g (5.2 mmol) of *N*-bromosccinimide. The reaction mixture was left stirring for 1 h, after which 20 ml of a saturated solution of NaHCO_3_ was added. Subsequently, the extraction was performed three times with diethyl ether (3 *x* 20 ml). The organic extracts were dried over Na_2_SO_4_, filtered, concentrated, and chromatographed. The title compound (1*S*,3*R*,8*R*)-2,2,16-tribromo-3,7,7,10-tetramethyltricyclo[6.4.0.0_1_,_3_]dodec-9-ene was obtained with a yield of 16% (18 mg, 0.4 mmol) and was recrystallized from n-pentane solution at room temperature.

### Refinement

All H atoms were fixed geometrically and treated as riding with C—H = 0.96 Å (methyl), 0.97 Å (methylene) and 0.98 Å (methine) with *U*_iso_(H) = 1.2 U_eq_(methylene, methine) or *U*_iso_(H) = 1.5 U_eq_(methyl).

### Crystal data

**Table:**

C_16_H_23_Br_3_	*F*(000) = 896
*M**_r_* = 455.07	*D*_x_ = 1.768 Mg m^−^^3^
Orthorhombic, *P*2_1_2_1_2_1_	Mo *K*α radiation, λ = 0.71073 Å
Hall symbol: P 2ac 2ab	Cell parameters from 3461 reflections
*a* = 9.2614 (5) Å	θ = 3.1–26.4°
*b* = 12.8215 (8) Å	µ = 7.07 mm^−^^1^
*c* = 14.3966 (11) Å	*T* = 180 K
*V* = 1709.52 (19) Å^3^	Prism, colourless
*Z* = 4	0.49 × 0.31 × 0.08 mm

### Data collection

**Table:**

Agilent Xcalibur (Sapphire1, long nozzle) diffractometer	3461 independent reflections
Graphite monochromator	3121 reflections with *I* > 2σ(*I*)
Detector resolution: 8.2632 pixels mm^-1^	*R*_int_ = 0.049
ω scans	θ_max_ = 26.4°, θ_min_ = 3.1°
Absorption correction: multi-scan (*CrysAlis PRO*; Agilent, 2010)	*h* = −11→11
*T*_min_ = 0.135, *T*_max_ = 1.000	*k* = −16→15
9721 measured reflections	*l* = −15→17

### Refinement

**Table:**

Refinement on *F*^2^	Hydrogen site location: inferred from neighbouring sites
Least-squares matrix: full	H-atom parameters constrained
*R*[*F*^2^ > 2σ(*F*^2^)] = 0.034	*w* = 1/[σ^2^(*F*_o_^2^) + (0.0387*P*)^2^ + 0.0947*P*] where *P* = (*F*_o_^2^ + 2*F*_c_^2^)/3
*wR*(*F*^2^) = 0.075	(Δ/σ)_max_ = 0.001
*S* = 1.04	Δρ_max_ = 0.66 e Å^−^^3^
3461 reflections	Δρ_min_ = −0.55 e Å^−^^3^
176 parameters	Extinction correction: *SHELXL97* (Sheldrick, 2008), Fc^*^=kFc[1+0.001xFc^2^λ^3^/sin(2θ)]^-1/4^
0 restraints	Extinction coefficient: 0.0034 (4)
Primary atom site location: structure-invariant direct methods	Absolute structure: Flack (1983), 1460 Friedel pairs
Secondary atom site location: difference Fourier map	Flack parameter: 0.012 (16)

### Special details

**Table:**

Experimental. Empirical absorption correction using spherical harmonics, implemented in SCALE3 ABSPACK scaling algorithm. CrysAlisPro (Agilent, 2010)
Geometry. All s.u.'s (except the s.u. in the dihedral angle between two l.s. planes) are estimated using the full covariance matrix. The cell s.u.'s are taken into account individually in the estimation of s.u.'s in distances, angles and torsion angles; correlations between s.u.'s in cell parameters are only used when they are defined by crystal symmetry. An approximate (isotropic) treatment of cell s.u.'s is used for estimating s.u.'s involving l.s. planes.
Refinement. Refinement of *F*^2^ against ALL reflections. The weighted *R*-factor *wR* and goodness of fit *S* are based on *F*^2^, conventional *R*-factors *R* are based on *F*, with *F* set to zero for negative *F*^2^. The threshold expression of *F*^2^ > 2σ(*F*^2^) is used only for calculating *R*-factors(gt) *etc*. and is not relevant to the choice of reflections for refinement. *R*-factors based on *F*^2^ are statistically about twice as large as those based on *F*, and *R*- factors based on ALL data will be even larger.

### Fractional atomic coordinates and isotropic or equivalent isotropic displacement parameters (Å^2^)

**Table:**

	*x*	*y*	*z*	*U*_iso_*/*U*_eq_	
C13	0.9676 (5)	0.5913 (4)	0.4115 (3)	0.0283 (12)	
H13A	0.9119	0.6220	0.4606	0.042*	
H13B	0.9989	0.5230	0.4299	0.042*	
H13C	0.9093	0.5861	0.3565	0.042*	
C1	1.1613 (4)	0.6641 (3)	0.2929 (3)	0.0110 (8)	
C2	1.0820 (5)	0.7571 (3)	0.3330 (3)	0.0164 (9)	
C3	1.0980 (5)	0.6589 (3)	0.3916 (3)	0.0160 (9)	
C4	1.2066 (6)	0.6600 (3)	0.4707 (3)	0.0251 (11)	
H4A	1.1567	0.6746	0.5285	0.030*	
H4B	1.2760	0.7154	0.4602	0.030*	
C5	1.2867 (6)	0.5560 (4)	0.4793 (3)	0.0280 (12)	
H5A	1.2275	0.5082	0.5151	0.034*	
H5B	1.3752	0.5675	0.5139	0.034*	
C6	1.3251 (5)	0.5037 (3)	0.3864 (3)	0.0233 (10)	
H6A	1.2356	0.4818	0.3573	0.028*	
H6B	1.3798	0.4410	0.4001	0.028*	
C7	1.4112 (5)	0.5671 (3)	0.3143 (3)	0.0196 (10)	
C8	1.3273 (4)	0.6703 (3)	0.2837 (3)	0.0146 (8)	
H8	1.3594	0.7261	0.3254	0.018*	
C9	1.3668 (4)	0.7037 (3)	0.1861 (3)	0.0186 (10)	
H9	1.4614	0.7250	0.1756	0.022*	
C10	1.2776 (5)	0.7050 (3)	0.1147 (3)	0.0158 (9)	
C11	1.1229 (5)	0.6707 (3)	0.1223 (3)	0.0161 (9)	
H11A	1.0988	0.6284	0.0687	0.019*	
H11B	1.0611	0.7318	0.1214	0.019*	
C12	1.0923 (4)	0.6086 (3)	0.2101 (3)	0.0139 (8)	
H12A	1.1318	0.5389	0.2041	0.017*	
H12B	0.9889	0.6028	0.2195	0.017*	
C14	1.5566 (5)	0.6008 (4)	0.3559 (4)	0.0427 (15)	
H14A	1.6119	0.5401	0.3719	0.064*	
H14B	1.5400	0.6419	0.4106	0.064*	
H14C	1.6088	0.6415	0.3111	0.064*	
C15	1.4416 (6)	0.4939 (4)	0.2310 (3)	0.0328 (13)	
H15A	1.4933	0.4336	0.2521	0.049*	
H15B	1.4985	0.5303	0.1856	0.049*	
H15C	1.3518	0.4727	0.2036	0.049*	
C16	1.3330 (6)	0.7378 (4)	0.0200 (3)	0.0286 (11)	
H16A	1.3172	0.6817	−0.0241	0.034*	
H16B	1.4361	0.7506	0.0236	0.034*	
Br1	0.89111 (5)	0.79294 (4)	0.28578 (3)	0.02481 (13)	
Br2	1.17768 (6)	0.88596 (3)	0.36490 (3)	0.02729 (14)	
Br3	1.23443 (7)	0.86490 (4)	−0.02407 (4)	0.04212 (18)	

### Atomic displacement parameters (Å^2^)

**Table:**

	*U*^11^	*U*^22^	*U*^33^	*U*^12^	*U*^13^	*U*^23^
C13	0.030 (3)	0.028 (3)	0.027 (3)	−0.002 (2)	0.016 (2)	0.009 (2)
C1	0.0118 (18)	0.0124 (18)	0.0088 (19)	−0.0009 (16)	0.0025 (16)	0.0028 (17)
C2	0.016 (2)	0.018 (2)	0.015 (2)	−0.0061 (18)	0.0004 (17)	−0.0025 (18)
C3	0.020 (2)	0.015 (2)	0.013 (2)	0.0007 (19)	0.0037 (18)	−0.0005 (17)
C4	0.047 (3)	0.021 (2)	0.007 (2)	0.003 (2)	−0.005 (2)	−0.0004 (18)
C5	0.046 (3)	0.026 (2)	0.013 (2)	0.008 (2)	−0.005 (2)	0.004 (2)
C6	0.032 (3)	0.019 (2)	0.020 (3)	0.007 (2)	−0.001 (2)	0.0051 (18)
C7	0.012 (2)	0.021 (2)	0.026 (3)	0.0040 (19)	−0.0045 (18)	0.0045 (19)
C8	0.0139 (19)	0.015 (2)	0.015 (2)	−0.0022 (17)	−0.0019 (19)	0.0036 (18)
C9	0.010 (2)	0.019 (2)	0.026 (3)	−0.0017 (18)	0.0026 (16)	0.007 (2)
C10	0.021 (2)	0.0145 (19)	0.012 (2)	0.0047 (19)	0.0061 (17)	0.0031 (17)
C11	0.020 (2)	0.018 (2)	0.010 (2)	−0.0006 (18)	−0.0006 (17)	−0.0011 (17)
C12	0.0130 (19)	0.0158 (19)	0.013 (2)	0.0010 (18)	0.0010 (18)	−0.0032 (18)
C14	0.020 (3)	0.042 (3)	0.066 (4)	−0.003 (2)	−0.022 (3)	0.018 (3)
C15	0.029 (3)	0.030 (3)	0.040 (3)	0.015 (2)	0.009 (2)	0.010 (2)
C16	0.033 (3)	0.030 (3)	0.023 (3)	0.010 (2)	0.011 (2)	0.012 (2)
Br1	0.0178 (2)	0.0254 (2)	0.0312 (3)	0.0070 (2)	0.0019 (2)	−0.0009 (2)
Br2	0.0377 (3)	0.0147 (2)	0.0295 (3)	−0.0026 (2)	−0.0072 (2)	−0.0044 (2)
Br3	0.0431 (3)	0.0365 (3)	0.0467 (4)	0.0046 (3)	0.0065 (3)	0.0243 (3)

### Geometric parameters (Å, º)

**Table:**

C13—C3	1.514 (6)	C7—C15	1.549 (6)
C13—H13A	0.9600	C7—C8	1.596 (6)
C13—H13B	0.9600	C8—C9	1.515 (6)
C13—H13C	0.9600	C8—H8	0.9800
C1—C2	1.516 (6)	C9—C10	1.318 (6)
C1—C12	1.528 (5)	C9—H9	0.9300
C1—C3	1.540 (6)	C10—C11	1.503 (6)
C1—C8	1.544 (5)	C10—C16	1.517 (6)
C2—C3	1.523 (6)	C11—C12	1.520 (6)
C2—Br2	1.930 (4)	C11—H11A	0.9700
C2—Br1	1.949 (4)	C11—H11B	0.9700
C3—C4	1.519 (6)	C12—H12A	0.9700
C4—C5	1.532 (6)	C12—H12B	0.9700
C4—H4A	0.9700	C14—H14A	0.9600
C4—H4B	0.9700	C14—H14B	0.9600
C5—C6	1.538 (6)	C14—H14C	0.9600
C5—H5A	0.9700	C15—H15A	0.9600
C5—H5B	0.9700	C15—H15B	0.9600
C6—C7	1.541 (6)	C15—H15C	0.9600
C6—H6A	0.9700	C16—Br3	1.973 (4)
C6—H6B	0.9700	C16—H16A	0.9700
C7—C14	1.535 (6)	C16—H16B	0.9700
			
C3—C13—H13A	109.5	C14—C7—C8	107.5 (4)
C3—C13—H13B	109.5	C6—C7—C8	111.8 (3)
H13A—C13—H13B	109.5	C15—C7—C8	112.1 (4)
C3—C13—H13C	109.5	C9—C8—C1	109.5 (3)
H13A—C13—H13C	109.5	C9—C8—C7	111.9 (3)
H13B—C13—H13C	109.5	C1—C8—C7	114.7 (3)
C2—C1—C12	117.5 (3)	C9—C8—H8	106.7
C2—C1—C3	59.8 (3)	C1—C8—H8	106.7
C12—C1—C3	122.7 (3)	C7—C8—H8	106.7
C2—C1—C8	118.3 (4)	C10—C9—C8	125.1 (4)
C12—C1—C8	112.0 (3)	C10—C9—H9	117.4
C3—C1—C8	117.4 (4)	C8—C9—H9	117.4
C1—C2—C3	60.9 (3)	C9—C10—C11	122.5 (4)
C1—C2—Br2	122.8 (3)	C9—C10—C16	119.5 (4)
C3—C2—Br2	122.1 (3)	C11—C10—C16	118.0 (4)
C1—C2—Br1	119.5 (3)	C10—C11—C12	113.0 (3)
C3—C2—Br1	118.5 (3)	C10—C11—H11A	109.0
Br2—C2—Br1	107.3 (2)	C12—C11—H11A	109.0
C13—C3—C4	113.1 (4)	C10—C11—H11B	109.0
C13—C3—C2	120.0 (4)	C12—C11—H11B	109.0
C4—C3—C2	118.1 (4)	H11A—C11—H11B	107.8
C13—C3—C1	120.2 (4)	C11—C12—C1	109.1 (3)
C4—C3—C1	116.1 (4)	C11—C12—H12A	109.9
C2—C3—C1	59.3 (3)	C1—C12—H12A	109.9
C3—C4—C5	111.9 (4)	C11—C12—H12B	109.9
C3—C4—H4A	109.2	C1—C12—H12B	109.9
C5—C4—H4A	109.2	H12A—C12—H12B	108.3
C3—C4—H4B	109.2	C7—C14—H14A	109.5
C5—C4—H4B	109.2	C7—C14—H14B	109.5
H4A—C4—H4B	107.9	H14A—C14—H14B	109.5
C4—C5—C6	114.9 (4)	C7—C14—H14C	109.5
C4—C5—H5A	108.5	H14A—C14—H14C	109.5
C6—C5—H5A	108.5	H14B—C14—H14C	109.5
C4—C5—H5B	108.5	C7—C15—H15A	109.5
C6—C5—H5B	108.5	C7—C15—H15B	109.5
H5A—C5—H5B	107.5	H15A—C15—H15B	109.5
C5—C6—C7	118.4 (4)	C7—C15—H15C	109.5
C5—C6—H6A	107.7	H15A—C15—H15C	109.5
C7—C6—H6A	107.7	H15B—C15—H15C	109.5
C5—C6—H6B	107.7	C10—C16—Br3	111.2 (3)
C7—C6—H6B	107.7	C10—C16—H16A	109.4
H6A—C6—H6B	107.1	Br3—C16—H16A	109.4
C14—C7—C6	109.9 (4)	C10—C16—H16B	109.4
C14—C7—C15	108.2 (4)	Br3—C16—H16B	109.4
C6—C7—C15	107.2 (4)	H16A—C16—H16B	108.0
